# Unveiling Salt Tolerance Mechanisms in Plants: Integrating the KANMB Machine Learning Model With Metabolomic and Transcriptomic Analysis

**DOI:** 10.1002/advs.202417560

**Published:** 2025-04-26

**Authors:** Shoukun Chen, Hao Zhang, Shuqiang Gao, Kunhui He, Tingxi Yu, Shang Gao, Jiankang Wang, Huihui Li

**Affiliations:** ^1^ State Key Laboratory of Crop Gene Resources and Breeding, Institute of Crop Sciences Chinese Academy of Agricultural Sciences (CAAS) Beijing 100081 China; ^2^ Nanfan Research Institute CAAS Sanya Hainan 572024 China; ^3^ Guangxi Key Laboratory of Rice Genetics and Breeding Rice Research Institute, Guangxi Academy of Agricultural Sciences Nanning Guangxi 530007 China

**Keywords:** KANMB, metabolomic, salt tolerance, *Spartina alterniflora*, transcriptomic

## Abstract

Salt stress presents a substantial threat to cereal crop productivity, especially in coastal agricultural regions where salinity levels are high. Addressing this challenge requires innovative approaches to uncover genetic resources that support molecular breeding of salt‐tolerant crops. In this study, a novel machine learning model, KANMB is introduced, designed to analyze integrated multi‐omics data from the natural halophyte *Spartina alterniflora* under various NaCl concentrations. Using KANMB, 226 metabolic biomarkers significantly linked to salt stress responses, grounded in metabolomic and transcriptomic profiles are identified. These biomarkers correlate with metabolic pathways associated with salt tolerance, providing insight into the underlying biochemical mechanisms. A co‐expression analysis further highlights the *MYB* gene *SaMYB35* as a pivotal regulator in the flavonoid biosynthesis pathway under salt stress. When overexpressed *SaMYB35* in rice (ZH11) grown under high salinity, it triggers the upregulation of key flavonoid biosynthetic genes, elevates flavonoid content, and enhances salt tolerance compared to wild‐type plants. The findings from this study offer a valuable genetic toolkit for breeding salt‐tolerant cereal varieties and demonstrate the power of machine learning in accelerating biomarker discovery for stress resilience in non‐model plant species.

## Graphical abstract

Salt stress endangers coastal cereal crops, requiring resilient crop solutions. This study employs machine learning (KANMB) to analyze multi‐omics data from halophyte *Spartina alterniflora*, revealing 226 salt‐stress biomarkers and linking them to tolerance pathways. The MYB gene SaMYB35 regulates flavonoid biosynthesis under salinity. Its overexpression in rice boosts flavonoid levels and salt tolerance, providing genetic resources for breeding climate‐resilient cereals.

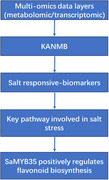

## 1 Introduction

The excessively high soil salinity poses a constant threat to the sustainability of crop production worldwide. Approximately 20% to 30% of the globally irrigated land is affected by soil salinization, and this proportion continues to grow due to land clearing and agricultural irrigation.^[^ ^1^ ^]^ As the population continues to grow, so does the demand for food.^[^ ^2^ ^]^ Consequently, breeding salt‐tolerant crops will not only enhance crop yields on salt‐affected farmlands but also expand the area of land available for cultivation, thereby effectively improving global food security. Halophytes, renowned for their inherent salt tolerance, exhibit significant potential in rehabilitating salt‐contaminated soils through various strategies, including phytoremediation, thereby fostering plant growth in saline environments.

*Spartina alterniflora*, a halophilic saltmarsh cordgrass in the Poaceae family, stands out as a prime example of exo‐recretohalophytes. It possesses specialized bicellular salt glands capable of secreting over 90% of absorbed Na⁺ and Cl⁻ ions, with gland densities exceeding 500 glands/cm^2^ on leaf surfaces.^[^ ^3^ ^]^ Additionally, its roots selectively absorb ions via voltage‐gated K⁺ channels and SOS1‐type Na⁺/H⁺ antiporters, maintaining a 20‐fold higher K⁺/Na⁺ ratio in shoots under 500 mm NaCl stress than glycophytes.^[^ ^4^ ^]^ *S. alterniflora* also accumulates osmoprotectants such as proline (up to 15 µmol g^−1^ FW) and glycine betaine, while exhibiting three‐ to fivefold higher antioxidant enzyme activities (e.g., superoxide dismutase [SOD] and catalase [CAT]) compared to salt‐sensitive plants.^[^ ^5^ ^]^ Coexisting with staple crops like rice (*Oryza sativa*), maize (*Zea mays*), and sorghum (*Sorghum bicolor*) in coastal wetlands, *S. alterniflora* efficiently crystallizes excess salt on its leaf surface, enabling survival in saline soils with conductivity exceeding 20 dS m^−1^.^[^ ^6^ ^]^ Its robust salt‐tolerance mechanisms make it a valuable genetic reservoir for improving stress resilience in cereals through gene mining and metabolic engineering.^[^ ^6^ ^]^ Previous studies, notably those involving the ectopic expression of genes like *SaHKT2;4*, *SaADF2*, *SaSRP3‐1*, *SaβNAC*, *SaARF*, *SaSce9*, and *SaVHAc1* in plants, have convincingly demonstrated the promising potential of genes derived from *S. alterniflora* in bolstering salt tolerance and increasing yield.^[^ ^3^, ^7^ ^]^ It is clear that genes from *S. alterniflora* exhibit advantages in improving crop salt tolerance and yield.

Biomarkers serve as precise indicators of physiological activity and metabolic status, enabling the definition of changes or differences in plants associated with specific environmental adaptations.^[^ ^8^ ^]^ Metabolites function as key biomarkers in stress response regulation.^[^ ^9^ ^]^ Among them, flavonoids such as quercetin and naringenin mitigate heavy metal toxicity and enhance salt tolerance by modulating ion homeostasis, osmotic balance, and reactive oxygen species (ROS) scavenging.^[^ ^10^ ^]^ For example, maize roots secrete flavonoids to alleviate aluminum toxicity,^[^ ^11^ ^]^ while quercetin accumulation in salt‐stressed tomatoes (*Solanum lycopersicum*) improves chlorophyll and carotenoid levels by reducing Na⁺/K⁺ ratios and ROS accumulation.^[^ ^12^ ^]^ Beyond flavonoids, other metabolites also contribute synergistically. Proline supplementation enhances root architecture in salt‐stressed rice,^[^ ^13^ ^]^ tryptophan (TRP) seed pretreatment improves root quality in sugar beet (Beta vulgaris) by regulating K⁺, Na⁺, and α‐amino‐N levels,^[^ ^14^ ^]^ and α‐lipoic acid (LA) foliar application boosts antioxidant defenses (e.g., increased cysteine, peroxidase [POD], and CAT activity) while reducing lipid peroxidation in salt‐stressed *Brassica napus*.^[^ ^15^ ^]^ Collectively, these findings suggest that plants employ a multi‐metabolite strategy—encompassing flavonoids, amino acids, and antioxidants—to coordinate ion regulation, osmoprotection, and oxidative stress mitigation, thereby enhancing salinity resilience across species.

While standard laboratory techniques, such as Western blotting, Matrix‐Assisted Laser Desorption/Ionization‐Time of Flight (MALDI‐TOF), and Enzyme‐Linked Immunosorbent Assay (ELISA), are routinely used to detect and analyze protein biomarkers and their corresponding genes in plant tissues, significantly advancing our understanding of plant biology.^[^ ^16^ ^]^ The development and application of these techniques demand considerable labor, materials, and financial resources. Rapid advances in omics approaches, such as genomics, proteomics, metabolomics, and transcriptomics, have facilitated the establishment of more comprehensive and insightful framework, enabling precise identification of biomarkers of plant stress states.^[^ ^17^ ^]^ Despite these advancements, the identification of stress‐related metabolic biomarkers in plants has remained challenging, hindering efforts to improve crop production. This is particularly true for specific plant species like *S. alterniflora*, which exhibits unique salt stress responses. Identifying biomarkers that can precisely indicate the salt stress response in this species is crucial for understanding its adaptation mechanisms and developing strategies to enhance its resilience in coastal ecosystems. Machine learning (ML) approaches underscored its significance in plant molecular biology, providing technical support for the discovery of regulatory pathways and unknown gene functions. For example, researchers have successfully elucidated the key metabolic pathways and core genes of *Microalga dunaliella* under salt stress, utilizing advanced techniques such as RNA‐seq meta‐analysis, systems‐level analysis, and supervised ML algorithms.^[^ ^18^ ^]^ AsmiR, a ML model that leverages Support Vector Machine (SVM) and K‐tuple nucleotide features, is tailored to predict miRNAs responsive to salt stress with exceptional accuracy, surpassing deep learning (DL) models in this particular task.^[^ ^19^ ^]^ mlDNA is an ML‐powered R package that facilitates the efficient identification of stress‐related genes in *Arabidopsis* transcriptome analysis.^[^ ^20^ ^]^ In addition, studies have employed ML methods to identify numerous genomic, transcriptomic, and proteomic salt tolerance‐related markers in *S. alterniflora*, including *High‐affinity potassium transporter* (*HKT*), *Sodium/hydrogen antiporter* (*NHX*), and *sugars will eventually be exported transporter* (*SWEET*) genes, demonstrating the analytical power of ML for mining stress‐related biomarkers.^[^ ^3^, ^21^ ^]^ Despite the remarkable achievements in utilizing ML for gene mining, the metabolites involved in mechanisms of salt tolerance in *S. alterniflora* have not been well‐defined, and metabolic biomarkers of plant response to saline conditions, which should be abundant in this species, are still lacking. Kolmogorov‐Arnold Network (KAN) is a novel DL model that has demonstrated great potential and advantages in fields such as time series, graph structures, and convolutions. Compared with traditional Multi‐Layer Perceptrons (MLPs), the core feature of KAN networks lies in placing the activation functions at the edges of the network (on the weights), and these activation functions are learnable, often parameterized using B‐splines. Thus, utilizing KAN for the identification of metabolic biomarker in *S. alterniflora* is an effective approach.

In this study, we developed a ML model, KANMB (**K**olmogorov‐**A**rnold **N**etwork for identifying **M**etabolic **B**iomarkers), tailor‐made for identifying metabolic biomarkers. By applying KANMB to large‐scale transcriptomic and metabolomic datasets generated from the halophyte *S. alterniflora* under varied salt concentrations, we successfully identified key metabolic biomarkers, including candidates within flavonoid biosynthesis, a pathway critical for salt tolerance. This innovative approach makes the KANMB model exceptionally precise and applicable in research endeavors aimed at unraveling the metabolic effects of salt stress on *S. alterniflora*. The set of candidate biomarkers is then refined by correlation analysis with salt stress, and we validate the most promising candidates through heterologous expression in rice followed by analysis of the resulting salt tolerance phenotype. The integration of the KANMB model with metabolomic and transcriptomic data has provided new insights into the salt tolerance mechanisms of *S. alterniflora*, and has the potential to guide future breeding efforts for salt‐tolerant crops.

## 2 Results

### 2.1 Workflow of KANMB for Identifying Metabolic Biomarkers

Traditional approaches for identifying and validating metabolic biomarkers have proven exceptionally challenging due to the complexity involved, often hindered by intricate experimental protocols and time‐consuming manual analyses, leading to low efficiency and highly susceptible outcomes influenced by various factors. KAN networks excel in achieving higher accuracy and interpretability in tasks like complex function fitting and solving partial differential equations. In the realm of time series prediction, KAN networks are capable of delivering more precise prediction outcomes by learning the intricate relationships and nonlinear patterns embedded within the data.

**Figure 1:**
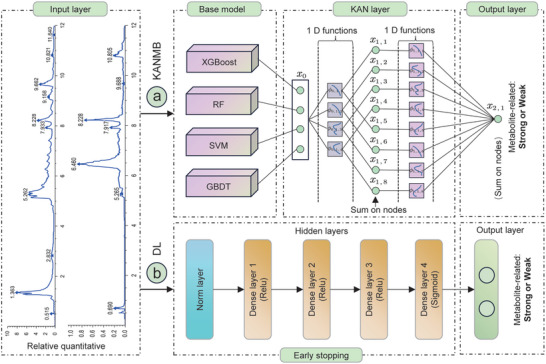
Overview of the KANMB and DL frameworks. Input data consists of the relative quantitative value of metabolites, and they were used as the input data module. a) The KANMB framework: The model integrates four base machine learning models—XGBoost, RF, SVM, and GBDT—along with a Kolmogorov‐Arnold Network (KAN) layer. The base models process metablote data, and their outputs are further refined through the KAN layer, which consists of an input layer, a hidden layer, and an output layer.Each KAN node applies learnable 1D activation functions to adaptively represent input features. b) Deep learning (DL) framework: The binary classification DL model comprises six layers—input, normalization, and three fully connected layers—culminating in an output layer. Detailed methodological descriptions are provided in the Methods section.

In this study, we developed KANMB, a Kolmogorov‐Arnold Network‐based (KAN) framework for metabolic biomarker identification (**Figure** **1**a). The KANMB layer consists of an input layer and an output layer, with parameters k = 1 and grid = 8, and utilizes a learnable node to dynamically adjust the weights of the features in the new feature matrix. The nodes in the KANMB perform iterative learning, allowing the learned functions to adaptively represent the input features. Initially, we transformed the relative quantitative values of 678 metabolites into a matrix containing 36 features. This matrix was then input into four machine learning models—Gradient Boosting Decision Tree (GBDT), SVM, Random Forest (RF), and eXtreme Gradient Boosting (XGBoost) —for prediction. The probability outputs from these models were combined into a new data matrix (678 metabolites × 4 features), which was subsequently process by the KAN model. Through iterative adjustments, the KAN model refined the weight distribution of these probability outputs to generate final classification prediction. The accuracy of identified metabolic biomarkers was validated through wet lab experiments. Notably, KANMB can also analyze gene expression profiles to identify key genetic metabolic markers. Additionally, we constructed a deep‐learning model for metabolic biomarkers identification and compared its performance with KANMB (Figure 1b).

### 2.2 Morphological and Physiological Responses of *S. alterniflora* to Salt Stress

**Figure 2:**
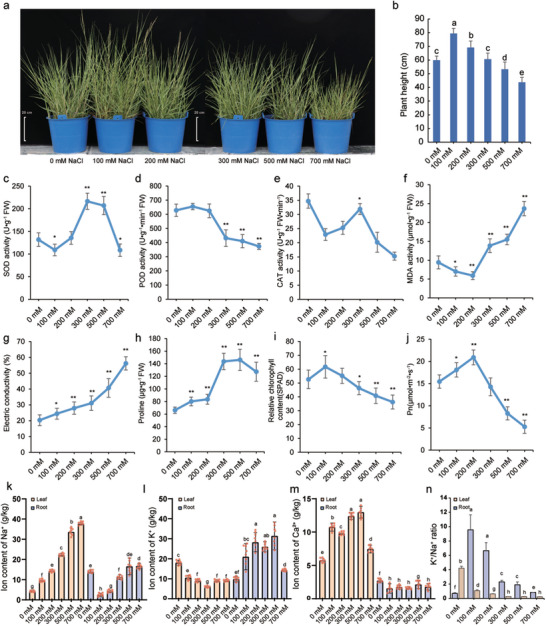
Impact of salt stress on growth and ion content of *S. alterniflora*. a) Growth phenotypes of *S. alterniflora* under different salt concentration stresses, Bar = 20 cm. b) Statistics of plant height under various salt stress concentrations (n = 15). c–j) Effects of varying NaCl concentrations on the physiological and biochemical characteristics of *S. alterniflora*, including (c) SOD, (d) POD, (e) CAT, (f) MDA, (g) electric conductivity, (h) proline content, (i) relative chlorophyll content, and (j) photosynthetic rate. (k‐m) Ion content in leaves and roots under different salt stress conditions: k) Na⁺, l) K⁺, and (m) Ca^2^⁺ levels (n = 8). n) K^+^/Na^+^ ratio in roots and leaves across various salt stress conditions. All values in the graphs are represented as means ± standard errors, with significant differences denoted by different letters (*p* < 0.05).

To investigate morphological changes in *S. alterniflora* associated with salt stress, we treated two‐week‐old plants with NaCl at a range of concentrations (0, 100, 200, 300, 500, and 700 NaCl) in greenhouse conditions. After two months of continuous treatment, examination of plant morphology indicated that plants treated with 100 mm NaCl exhibited the most vigorous overall growth among treatment groups, with overall growth gradually declining with increasing salt concentration, with the strongest growth inhibition observed at 700 mm NaCl (**Figure** **2**a,b).

Further analysis of physiological and biochemical responses of *S. alterniflora* to varying levels of salt stress revealed that activity of stress‐related enzymes, superoxide dismutase (SOD) highest at 300 mm NaCl, significantly greater than controls (*P* = 3.65e^−11^) and lowest at 100 mm NaCl concentrations (*P* = 9.11e^−4^), which were significantly lower than that observed in untreated control plants (Figure 2c). Peroxide dismutase (POD) activity peaked at 100 mm NaCl, not significantly different from controls (Figure 2d), gradually decreasing until to 700 mm (*P* = 3.16e^−13^). Catalase (CAT) activity followed a similar pattern to that of SOD (Figure 2e), while the stress‐related parameters of malondialdehyde (MDA) activity, electrical conductivity, and free proline content gradually increased along with salt concentration (Figure 2f–h), suggesting increased oxidative stress, membrane damage, and adaptation to osmotic stress, respectively. Conversely, chlorophyll content and photosynthetic rate (Pn) peaked at 200 mm, then declined at higher salt levels (700 mm, *P* = 1.73e^−12^), suggesting impaired photosynthesis under high saline conditions (Figure 2i,j). These results implied that *S. alterniflora* exhibited the strongest adaptive response to saline at 100 mm NaCl.

To gain further insights into the ion transport mechanisms in *S. alterniflora* under salt stress, we investigated the behavior of Na^+^, K^+^, and Ca^2+^, which are known to significantly influence salt stress responses. Detection of ion contents in *S. alterniflora* root and leaf tissues under varying salt conditions revealed that Na^+^ contents were lowest in roots at 100 mm (*P* = 1.22e^−5^) and highest at 700 mm (*P* = 0.037), while K^+^ increased with salt concentrations, peaking at 500 mm (*P* = 3.56e^−3^), and no significant difference in Ca^2+^ levels was observed between any treatment groups and controls (Figure 2k–m). In leaf samples collected after two months of salt exposure, Na^+^ concentration increased along with treatment concentration, peaking at 700 mm. By contrast, K^+^ levels decreased, reaching their lowest point at 300 mm NaCl (*P* = 1.17e^−6^). Leaf Ca^2+^ showed no obvious trend between NaCl levels, but was highest at 500 mm (*P* = 1.76e^−7^) compared to 0 mm NaCl (Figure 2k–m). The K^+^/Na^+^ ratio in root tissue was highest at 100 mm (*P* = 1.82e^−3^), but steadily declined in leaves with increasing salt exposure (Figure 2n). These results demonstrate that salt stress exerts a pronounced effect on ion contents in the root and leaf tissues of *S. alterniflora*, with particularly notable changes observed in Na^+^ and K^+^ levels. These findings provide insights into the adaptive mechanisms of *S. alterniflora* under salt stress.

### 2.3 Investigating the Adaptability of *S. alterniflora* to High Salt Stress Through Integrated Metabolomics and Transcriptomics Analysis

**Figure 3:**
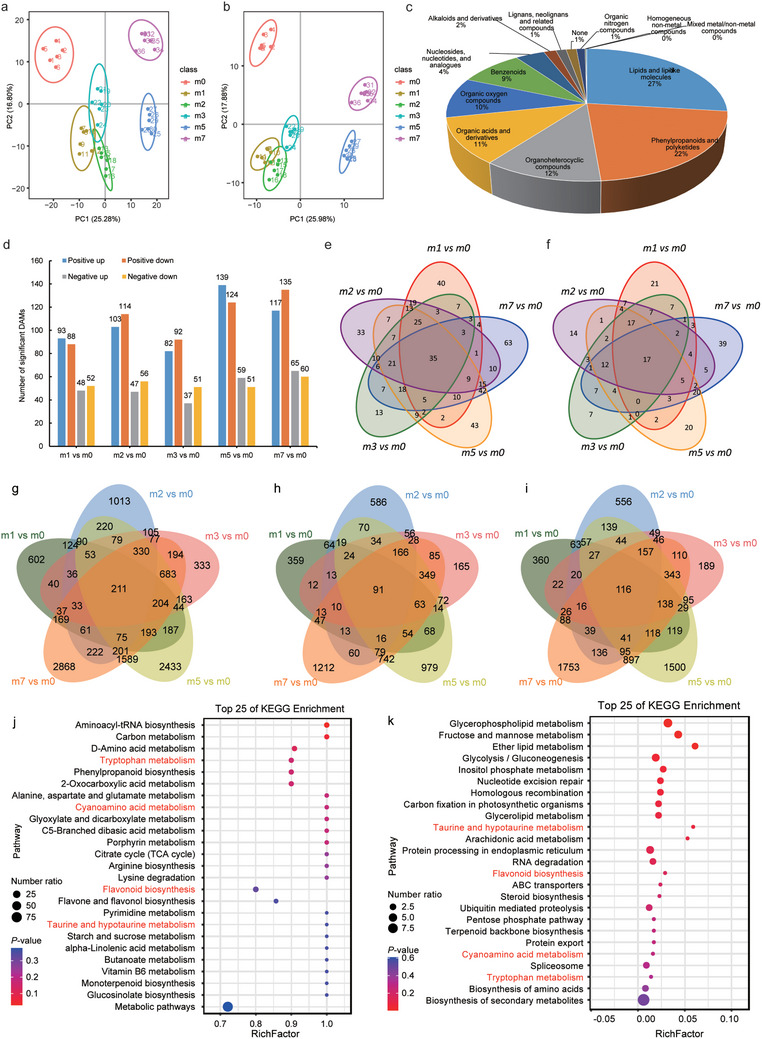
Summary of metabolome and transcriptome data sets. a) PCA analysis of metabolites in all samples in positive ion mode; b) PCA analysis of metabolites in all samples in negative ion mode. In the figure, the horizontal axis PC1 and vertical axis PC2 represent the scores of the first and second principal components, respectively. Scatter points of different colors represent samples from distinct experimental groups, while the ellipses depict the 95% confidence intervals. c) Primary classification of total metabolites in both positive and negative ion modes; d) Statistics of differential metabolites in *S. alterniflora* across various concentration treatments, displaying both upregulated and downregulated metabolite counts in both ion modes; e) Venn diagram analysis of positive ion metabolites across different concentrations of NaCl; f) Venn diagram analysis of negative ion metabolites across different concentrations of NaCl. g) Venn diagram analysis of total DEGs under salt stress conditions; h) Venn diagram analysis of upregulated DEGs under salt stress conditions; i) Venn diagram analysis of downregulated DEGs under salt stress conditions. j) KEGG analysis of all DAMs under salt stress; k) KEGG analysis of all DEGs under salt stress. The red‐labeled pathways in the figure represent the shared KEGG pathways between the metabolome and transcriptome.

To uncover metabolites potentially contributing to *S. alterniflora* resilience against high salt stress, we performed a comprehensive metabolomics analysis on leaves collected from *S. alterniflora* plants that were cultivated for 60 days under controlled greenhouse conditions with the same range of NaCl concentrations as above. Principal component analysis (PCA) revealed obvious separation among treatment groups with reasonably tight clustering of replicates within groups (**Figure** **3**a,b). In total, 781 unique primary metabolites were identified across all samples (Figure 3c; Tables S1 and S2, Supporting Information), among which, lipids and lipid‐like molecules accounted for the largest proportion (26.89%), followed by phenylpropanoids and polyketides (21.77%), organoheterocyclic compounds (11.65%), and organic acids and derivatives (11.27%), with the remaining metabolites comprising <10% each. Trend analysis of metabolite accumulation across salt gradients revealed eight distinct expression clusters. Metabolites in clusters C3 and C7 exhibited sustained accumulation at high salinity (≥500 mm NaCl), peaking at 500 and 700 mm, respectively, suggesting their involvement in long‐term adaptation. In contrast, clusters C4 (130 metabolites) and C5 (91 metabolites) showed progressive upregulation at moderate salinity (200–300 mm), stabilizing thereafter, indicative of early‐stage stress responses. Clusters C8 (142 metabolites) and C1 (107 metabolites) displayed transient accumulation at 100 mm, suggesting roles in acute stress signaling, while cluster C6 (164 metabolites) showed progressive suppression with increasing salinity, implying functional inhibition. Notably, the dominant accumulation in C8, tightly correlated with salt intensity, suggests core metabolic signatures of *S. alterniflora* salt tolerance and potential biomarker candidates. Further analysis of differentially abundant metabolites (DAMs; including positive and negative ions) between each treatment group and untreated controls indicated that plants exposed to higher NaCl concentrations contained a greater number of DAMs, with plants grown under 500 and 700 mm conditions having more DAMs than other treatment groups, while the fewest DAMs were detected in plants treated with 300 mm NaCl (Figure 3d). Venn diagram analysis indicated that 35 total DAMs were obtained in positive ion detection mode, while 17 DAMs were detected in negative mode across all salt treatment groups (Figure 3e,f; Tables S3 and S4, Supporting Information).

To define salt stress‐associated transcriptomic changes in *S. alterniflora*, we conducted bulk RNA‐seq analysis of leaf samples from each NaCl treatment group. Differential expression analysis identified 12670 differentially expressed genes (DEGs), with 5564 upregulated and 7388 downregulated. The number of DEGs increased with salt concentration, peaking at 3028 upregulated and 4119 downregulated genes at 700 mm NaCl. Among 211 core DEGs (91 upregulated, 116 downregulated), three expression patterns emerged (Figure 3g–i; Figure S1, Supporting Information): i) transient responders peaking at 100 mm, ii) gradual modulators progressively changing across concentrations, and iii) late‐phase regulators peaking at ≥500 mm. Notably, gradual modulators dominated (55% of core DEGs), with a 40‐gene cluster (C8) exhibiting sustained suppression under prolonged high salinity, underscoring transcriptional reprogramming as a key adaptive strategy. Further GO functional annotation analysis with the NetGO 3.0 ML model showed that cellular homeostasis, ion homeostasis, metal ion homeostasis, and vacuolar membrane terms were all significantly enriched (*P* < 0.01) in up‐regulated, but not down‐regulated, DEGs. These results suggested that the majority of upregulated genes were related to transport processes (Figure S2, Supporting Information), which drew our attention to ion transporter response to saline conditions.

To better understand the regulatory networks controlling salt stress‐responsive metabolic changes in *S. alterniflora*, we conducted an integrated analysis of transcriptomic and metabolomic data. KEGG pathway enrichment analysis identified the top 25 most significantly enriched pathways in both DAMs (Figure 3j) and DEGs (Figure 3k) under salt stress conditions. Subsequent comparative analysis uncovered revealed four pathways that were present in the top 25 pathways of both DEGs and DAMs, including tryptophan metabolism, cyanoamino acid metabolism, flavonoid biosynthesis, and taurine and hypotaurine metabolism (Figure 3j,k). Notably, tryptophan metabolism, cyanoamino acid metabolism, and flavonoid biosynthesis have all been previously reported to play crucial roles in salt stress responses.^[^ ^22^ ^]^ These findings suggested that these pathways may be essential for adaptive response to salt stress in *S. alterniflora*.

### 2.4 Establishment of “Metabolite Versus Gene” Modules in *S. alterniflora* Under Salt Stress

**Figure 4:**
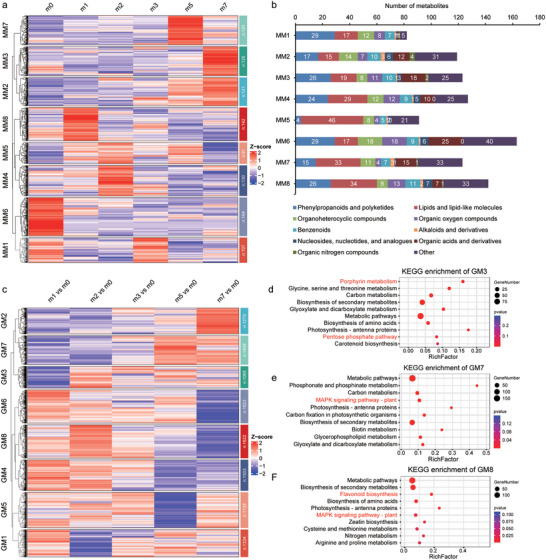
Association of dynamic metabolism of major metabolites with gene expression patterns under salt stress in *S. alterniflora*. a,c) Metabolite modules (MMs) versus gene modules (GMs). Accumulation patterns of metabolites in 8 MMs (a), and Log_2_FC values of 11 554 co‐expressed genes in 8 GMs (c) are shown. Z‐scores of data sets were standardized to −2 to 2. (b) Statistics of the class categories of metabolites in 8 MMs. d–f) KEGG analysis of co‐expressed genes in GM3 (d), GM7 (e), GM8 (f).

To further screen our above metabolomic and transcriptomic data for differentially regulated metabolites that could participate mediating tolerance to salt stress, we first performed Z‐score clustering to identify conjointly accumulating metabolites in our metabolomics data. This analysis identified eight such groups of co‐regulated metabolites, or metabolic modules (MMs; **Figure** **4**a). Specifically, MM6 comprised metabolites that were enriched at 0 mm NaCl, whereas metabolites in MM3 were primarily detected in samples from plants exposed to 700 mm NaCl (Figure 4a). We observed that metabolite distribution varied widely among MMs. For instance, lipids and lipid‐like molecules were highly enriched in MM5, while phenylpropanoids and polyketides were concentrated in MM1 and MM6, and organoheterocyclic compounds and organic oxygen compounds were predominantly found in MM6 (Figure 4b).

To then define the genetic basis of salt‐responsive metabolite accumulation patterns, we conducted metabolite co‐expression analyses with the 12670 DEGs (|Log_2_FC| ≥ 1 and *P* ≤ 0.05) obtained in our above RNA‐seq analysis. Calculation of Pearson correlation co‐efficient (PCC) values between gene transcript levels of DEGs and metabolite accumulation values identified 11554 total DEGs that were significantly correlated with at least one metabolite (|PCC| ≥ 0.8, Table S5, Supporting Information). Using the same clustering approach as for metabolites above, these 11554 DEGs were also grouped into 8 gene modules (GMs) based on their Log_2_FC values (Figure 4c), which further underscores the robust correlation between genes and metabolites in terms of their expression patterns.

Subsequent KEGG analysis of the 8 GMs revealed several significantly enriched metabolic pathways that were directly related to biosynthesis of the metabolites in corresponding MMs. For instance, the enriched MPs in GM3 included porphyrin metabolism, pentose phosphate pathway, and carotenoid biosynthesis (Figure 4d), which are responsible for the production of organic acids and derivatives as well as organic oxygen compounds, all of which consistently clustered into MM3. Moreover, phosphonate and phosphinate metabolites mainly clustered into MM3 and MM6, while corresponding phosphonate and phosphinate metabolism pathways were enriched in GM7 (Figure 4e). The enriched flavonoid biosynthesis pathways in GM8 corresponded to differentially accumulated flavonoids (i.e., phenylpropanoids and polyketides) that clustered in MM1, MM6, and MM8 (Figure 4f). In summary, these data indicated that major metabolic pathway dynamics largely paralleled the salt‐responsive gene expression pathways responsible for their regulation and biosynthesis in *S. alterniflora*.

We further used weighted gene co‐expression network analysis (WGCNA) to construct a co‐expression network of salt stress‐responsive genes in *S. alterniflora*, which revealed the classification of nine gene modules from among 12670 differentially expressed genes. Notably, the gray, blue, and green modules encompassed 3269, 2251, and 1068 genes respectively, constituting significant proportions of the DEGs in *S. alterniflora* under salt stress (Figure S3, Supporting Information). Genes within each module exhibited specific expression patterns, with gray module genes being highly expressed at low NaCl concentrations and blue module genes being upregulated at high NaCl concentrations. Further functional enrichment analysis revealed that genes in the gray module were predominantly associated with transmembrane transport, whereas those in the blue module were linked to α‐amino acid and glutamine metabolic processes. KEGG pathway analysis indicated that genes in the gray module were enriched in flavonoid biosynthesis pathways, whereas those in the blue module were associated with MAPK signaling pathways and amino acid metabolism. By integrating the enrichment results of DEGs and DAMs, we observed that salt stress significantly promoted the expression of genes related to flavonoid biosynthesis pathways, which are crucial for salt tolerance in *S. alterniflora*.

### 2.5 KANMB‐Based Selection of the Optimal Metabolite Biomarkers

**Figure 5:**
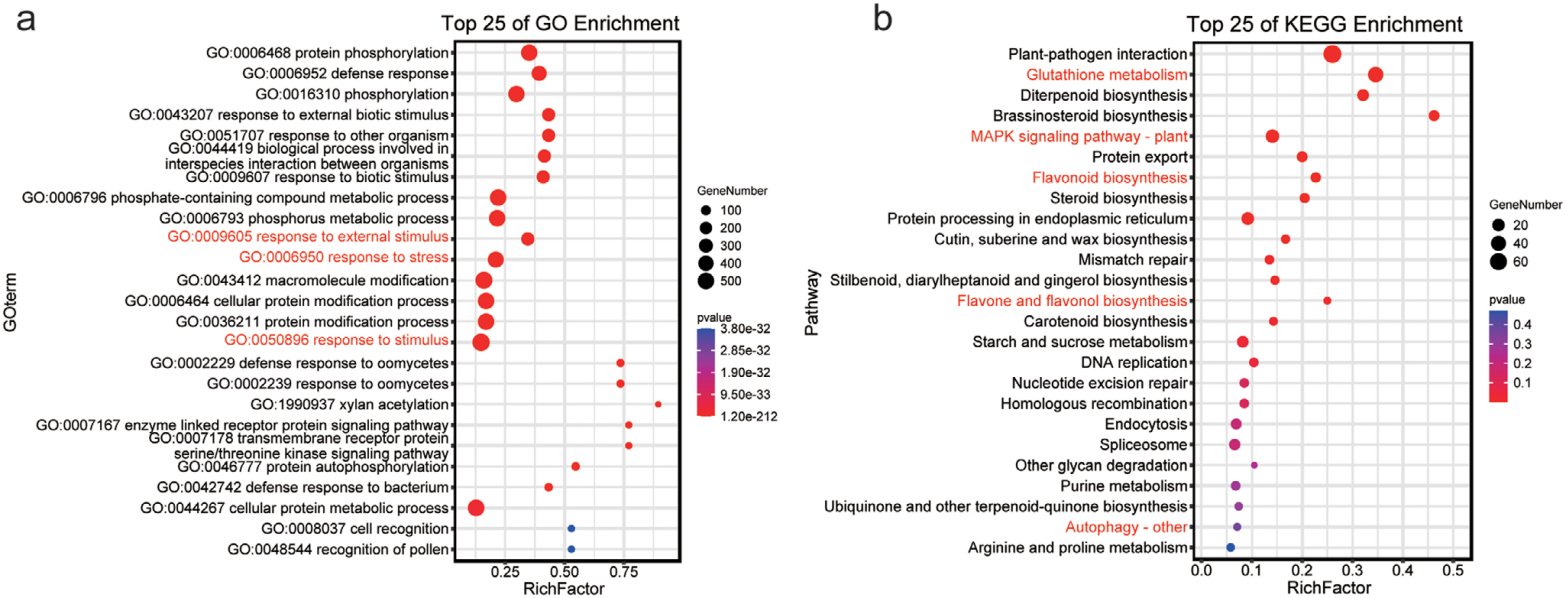
Functional enrichment analysis of DEGs corresponding to metabolic biomarkers identified via the KANMB module. a) GO analysis of DEGs corresponding to the biomarkers, where purple represents biological processes, green represents cellular components, and black represents molecular functions. b) KEGG analysis of DEGs associated with the identified biomarkers.

To identify metabolic biomarkers associated with salt stress, we utilized KANMB to analyze metabolomic expression data from *S. alterniflora* grown under a range of NaCl concentrations. The KANMB model identified 226 significant metabolites (prediction probability threshold ≥ 0.99; Table S6, Supporting Information). Among them, Phenylpropanoids and polyketides, as well as Lipids and lipid‐like molecules, exhibited the highest proportions. Further screening of the co‐expression modules identified 5737 DEGs corresponding to the 226 biomarkers, with a threshold of |Pearson correlation coefficient (PCC)| > 0.8. Subsequent GO functional annotation analysis using the NetGO 3.0 ML model revealed significant enrichment (*P* < 0.01) in DEGs related to stress response, response to external stimulus, response to stimulus (**Figure** **5**a). Additionally, KEGG analysis indicated enrichment of glutathione metabolism, MAPK signaling pathway, flavonoid biosynthesis, flavone and flavonol biosynthesis, and autophagy among these DEGs (Figure 5b). These findings suggest that these DEGs play crucial roles in the plant's ability to adapt and respond to salt stress, likely through the regulation of stress signaling and secondary metabolite biosynthesis.

To compare whether there are differences in screening results between KANMB and other ML methods, we concurrently applied four different machine learning approaches: SVM, XGBoost, RF, GBDT, and DL, for the identification of metabolic biomarkers. Performance metrics (Accuracy/Recall/AUC) demonstrated that KANMB achieved competitive results (0.92/0.77/0.86), surpassing RF (0.86/0.43/0.95) and GBDT (0.91/0.73/0.96) in Accuracy while slightly trailing DL (0.96/0.90/0.94) and XGBoost (0.90/0.63/0.97) in AUC. Notably, KANMB outperformed SVM (0.93/0.73/0.98) in Recall (0.77 vs 0.73) despite its lower AUC (Figure S4, Supporting Information). These findings suggest that while KANMB does not excel in all metrics, its balanced Accuracy and Recall, coupled with its focus on biological interpretability, make it a robust tool for identifying biologically relevant salt stress biomarkers.

The results indicated that, except for RF, which scored below 0.99 in screening the metabolic biomarkers, the SVM, XGBoost, GBDT, and DL other three models identified 114, 16, 217, and 144 biomarkers respectively (with a probability > 0.99). Further screening of co‐expression modules using SVM, XGBoost, GBDT, and DL SVM, DL, and XGBoost, respectively, revealed 1435, 1012, 9887, and 5789 DEGs corresponding to the identified biomarkers, with a threshold of |PCC| > 0.8. Subsequent GO and KEGG analyses of these DEGs showed that SVM, DL, and GBDT had similar enrichment patterns, primarily in GO terms related to protein phosphorylation (Figure S5, Supporting Information), including GO:0 006468 (protein phosphorylation) and GO:00 16310 (phosphorylation), as well as those associated with stress response, such as GO:0 006952 (defense response) and GO:00 50896 (response to stimulus). In contrast, the DEGs corresponding to XGBoost were predominantly enriched in stress response‐related terms, including GO:0 006952 (defense response), GO:0 006950 (response to stress), and GO:00 50896 (response to stimulus). Further KEGG enrichment analysis revealed that SVM and DL were mainly enriched in plant‐pathogen interaction, glutathione metabolism, and MAPK signaling pathway, while XGBoost was enriched in ABC transporters, sphingolipid metabolism, and aflatoxin biosynthesis, and GBDT was enriched in phenylpropanoid biosynthesis and plant hormone signal transduction (Figure S6, Supporting Information). Compared to the KAN method, the DEGs corresponding to the biomarkers identified by these four ML methods were not significantly enriched in pathways related to salt stress, such as flavonoid biosynthesis and flavone and flavonol biosynthesis. Despite KANMB's slightly lower AUC (0.86 vs XGBoost's 0.98), its emphasis on biologically relevant pathways confers a distinct advantage for metabolomic biomarker discovery under salt stress conditions.

### 2.6 Key Regulatory Factors in Flavonoid Accumulation

**Figure 6:**
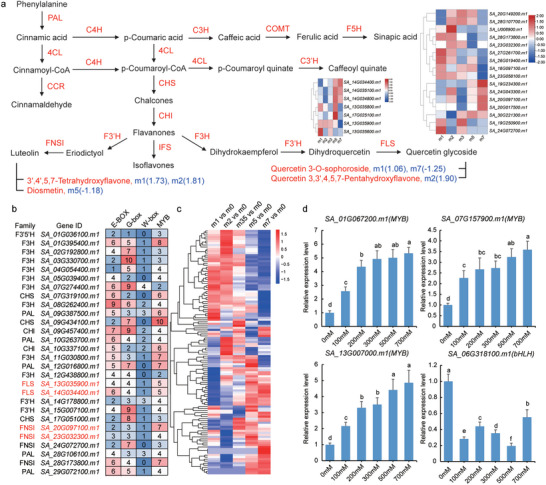
Regulation of flavonoid synthesis pathway genes in *S. alterniflora*. a) The biosynthetic pathway of major antioxidant components in *S. alterniflora*. The metabolites highlighted in red belong to luteolin or quercetin glycoside. The blue labels on the metabolites represent the Log_2_FC values relative to controls, while the red labels on the arrows indicate the synthase genes. b) Analysis of promoter binding sites for enzyme genes that are differentially expressed in at least four treatments. The color gradient from blue to red signifies a gradual increase in the number of binding sites. c) Expression profiles of differentially expressed *MYB* and *bHLH* genes in *S. alterniflora* under salt stress. The color gradient from blue to red represents increasing gene expression. d) Relative expression levels of four transcription factors after treatment with different concentrations of NaCl. Error bars indicate the standard error of the mean (SEM, n = 3). Different letters above the columns indicate significant differences at the *p* ≤0.05 level.

Based on the results above, which demonstrate that phenylpropanoid and polyketide flavonoids predominantly accumulate in all modules except MM5 (Figure 4b), and given that both DEGs, DAMs, and biomarker‐associated DEGs are linked to flavonoid biosynthesis, we subsequently investigated the metabolites involved in the flavonoid biosynthesis pathway (**Figure** **6**a).

To gain a deeper understanding of the role of flavonoid biosynthesis pathway in *S. alterniflora*, we conducted analysis on the genes involved in this pathway. Firstly, we identified 210 genes involved in flavonoid biosynthesis at the genome‐wide level, of which 118 genes displayed differential expression in response to at least one NaCl treatment (Table S7, Supporting Information). Our analysis highlighted three genes (*SA_17G051000.m1*, *SA_05G039400.m1*, *SA_20G097100.m1*) that consistently altered their expression across all treatments. Notably, *SA_17G051000.m1* (CHS) and *SA_20G097100.m1* (FNS) were upregulated across all salt concentrations, whereas *SA_05G039400.m1* (F3H) exhibited downregulation. Under high NaCl concentrations, a prominent downregulation trend was observed among most *PAL* genes, suggesting that this reduction in *PAL* gene expression may diminish the activity of crucial enzymes and, consequently, decrease the synthesis efficiency of precursor substances, leading to a decline in phenylalanine metabolism. Conversely, the majority of *F3H* and *FLS* genes were upregulated under salt stress, pointing toward a potential enhancement of flavonol metabolism through the promotion of key enzyme activities by *FLS* gene expression. Our results indicate that the expression of five *FLS* genes, namely *SA_13G035900.m1*, *SA_14G034400.m1*, *SA_14G035100.m1*, *SA_14G034800.m1*, and *SA_17G025100.m1*, was significantly upregulated under salt stress conditions. Similarly, the expression of five *FNSI* genes, specifically *SA_20G097100.m1*, *SA_23G032300.m1*, *SA_20G149200.m1*, *SA_26G019400.m1*, and *SA_20G017500.m1*, was also significantly upregulated in response to salt stress. In contrast, only two *FNSI* genes, *SA_28G173800.m1* and *SA_16G097100.m1*, showed significant downregulation at high NaCl concentrations (Table S7, Supporting Information). These findings suggest that salt stress affects the accumulation of flavonoids in *S. alterniflora* by altering the expression of genes involved in the flavonoid biosynthesis pathway, which aligns with previous research.

To further identify key TFs associated with flavonoid accumulation, co‐expression cluster analysis was conducted between the above flavonoid synthase genes and differentially expressed TFs from our RNA‐seq data (Table S8, Supporting Information), which yielded two distinct clusters of closely associated TFs and flavonol synthase‐encoding target genes (Figure S7, Supporting Information). Additionally, we inspected the distribution of *cis*‐acting promoter elements in flavonoid biosynthesis genes that were differentially expressed in at least four NaCl treatments. This analysis revealed that each gene harbored at least one E‐box and one G‐box motif, the former of which is recognized by bHLH TFs and both of which are recognized by bZIP TFs (Figure 6b). The majority of these flavonoid pathway genes also carried W‐boxes (recognized by WRKY TFs) and as well as at least two MYB TF binding sites. Heatmap analysis indicated that all bHLH and MYB TFs differentially expressed in response to salt exposure (Figure 6c). By setting a threshold of |Log_2_FC| ≥ 1 and FPKM > 5 across all NaCl treatments, we obtained a refined list of 15 TF DEGs, including seven *MYB* genes, four *bHLH* genes, three *WRKY* genes, and one *bZIP* gene. Notably, six *MYBs* and three *bHLHs* were significantly upregulated across all NaCl concentrations compared to the untreated controls, while one *MYB* (*SA_11G002700.m1*), one *bHLH* (*SA_06G318100.m1*), and one *bZIP* (*SA_08G296500.m1*) were downregulated under salt stress. Additionally, the three *WRKY* genes were upregulated under all treatments from 0 to 500 mm NaCl, but were downregulated at 700 mm. Validation of salt‐responsive expression by qRT‐PCR in four *MYBs* and one *bHLH* confirmed upregulation of the three *MYB* genes under increasing salt concentrations, and decreasing *bHLH* transcription with increasing NaCl levels (Figure 6d).

### 2.7 *SaMYB35* Positively Regulates Flavonoid Biosynthesis

**Figure 7:**
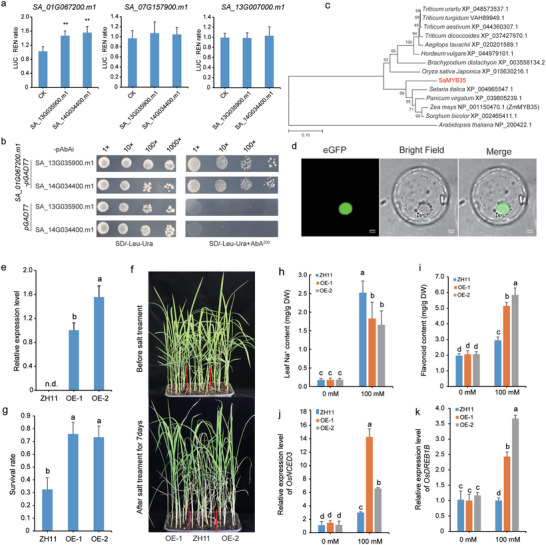
Overexpression of *SaMYB35* enhanced salt tolerance in rice. a) Activation of *FLS* promoters by SaMYB35 in dual‐luciferase reporter assays. b) Validation of the interaction between SaMYB35 and the promoters of two *FLS* genes through yeast one‐hybrid (Y1H) experiments. c) Phylogenetic analysis of SaMYB35 with MYB proteins from other grass species, using *Arabidopsis* NP_200 422.1 as an outgroup. (d) Subcellular localization of SaMYB35 protein. Bar = 10 µm. e) Relative expression levels of the *SaMYB35* gene in ZH11 and two transgenic lines. f) Phenotypic comparison of transgenic rice overexpressing *SaMYB35* before and after salt stress. ZH11 serves as the control, while OE‐1 and OE‐2 represent two independent overexpressing lines. g) Survival rate statistics of transgenic rice overexpressing *SaMYB35* after 7 days of salt stress exposure. h) Quantification of Na^+^ content in leaves of ZH11 and transgenic lines upon treatment with 0 and 100 mm NaCl. i) Flavonoid content measured under normal growth conditions and following treatment with 100 mm NaCl. Data are presented as mean ± SD (n = 6). j–k) Expression of the stress marker gene j) *OsNCED3* and k) *OsDREB1B* in ZH11 and transgenic lines under salt stress conditions. Different letters in the figures indicate statistical significance determined by Student's *t*‐tests (*p* < 0.01), n.d. indicated not detected.

We then conducted functional assays to verify that three *MYB* TFs indeed contributed to salt tolerance in *S. alterniflora* using two downstream *FLS* genes, *SA_13G035900.m1* and *SA_14G034400.m1*, as regulatory targets. For these dual‐luciferase assays in *N. benthamiana* leaves, the two *FLS* gene promoters were fused to a LUC‐reporter (i.e., pro*SA_13G035900.m1*::LUC and pro*SA_14G034400.m1*::LUC) and each MYB was cloned into an expression vector driven by a 35S promoter (35S::*SA_01G067200.m1*, 35S::*SA_07G157900.m1*, and 35S::*SA_13G007000.m1*) to serve as effectors. Following co‐transfection, we observed that *SA_01G067200.m1* could activate transcription of the *SA_13G035900.m1* and *SA_14G034400.m1* promoters to generate LUC signal, whereas *SA_07G157900.m1* and *SA_13G007000.m1* had no effect on these promoters (**Figure** **7**a). Subsequent Y1H assays further demonstrated that SA_01G067200.m1 could interact with the *SA_13G035900.m1* and *SA_14G034400.m1* promoter fragments in yeast (Figure 7b).

Phylogenetically, the SA_01G067200.m1 exhibits the strongest homology to the maize ZmMYB35, characterized by the presence of two complete MYB domains and belonging to the R2R3‐MYB subfamily (Figure 7c). Consequently, we have designated the *SA_01G067200.m1* gene as *SaMYB35*. Subcellular localization indicated that SaMYB35 protein is mainly expressed in the nuclear (Figure 7d). To validate its role in salt stress response, we transformed rice line ZH11 with *SaMYB35* to determine whether its heterologous expression could enhance salt tolerance. Two independent transgenic lines, OE‐1 and OE‐2, exhibiting high expression levels, were chosen for further subsequent analysis (Figure 7e). Our results revealed that transgenic overexpression of the *SaMYB35* gene could indeed enhance salt tolerance in rice (Figure 7f–g), the survival rate of the transgenic lines following salt stress was significantly higher than that of ZH11 (*P* < 0.05), and the Na^+^ content revealed that the transgenic plants maintained lower Na^+^ levels in their leaves under salt stress compared to ZH11 (Figure 7h). In addition, results indicated that flavonoid contents were significantly increased in transgenic rice plants grown under 100 mm NaCl conditions compared with that in ZH11 (Figure 7i), and salt stress induced the expression of the salt stress marker gene, *OsNCED3* and *OsDREB1B*, in two transgenic lines (Figure 7j,k). We further validated the salt tolerance function of *SaMYB35* using a yeast heterologous expression system. Under salt stress, yeast strains expressing *SaMYB35* exhibited significantly enhanced growth compared to control strains, demonstrating its functional role in promoting salt tolerance (Figure S8, Supporting Information). Taken together, these results identified the flavonoid biosynthesis MYB TF, *SaMYB35*, as critical component of salt tolerance in *S. alterniflora*.

## 3 Discussion

### 3.1 The KANMB Outperforms Traditional Methods in Deciphering Complex Biological Process

Traditional approaches for identifying and validating metabolic biomarkers have made the precise recognition of these markers an exceptionally daunting task. Encumbered by intricate experimental protocols and time‐consuming manual analysis, these methods suffer from low efficiency and outcomes that are susceptible to various influencing factors. Although the integration of chemistry and biotechnology has significantly facilitated the detection and separation of metabolites, the subsequent intricate in‐depth analysis still presents formidable hurdles.^[^ ^23^ ^]^ Research has demonstrated that under salt stress, significant alterations occur in the biosynthesis of sugars, amino acids, polyamines, organic acids, aromatic compounds, and plant hormones in rice, however, the challenge remains to identify from this myriad of altered biomarkers those that can act as modulators or substrate binding agents specifically for salt‐sensitive rice varieties.^[^ ^24^ ^]^

To address these limitations, we innovatively developed a model named KANMB specifically for biomarker identification. KANMB harnesses the groundbreaking neural network architecture of KAN, which distinguishes itself from conventional neural networks by incorporating learnable activation functions at the network's edges, rather than adhering to fixed activation functions. This distinctive feature grants KANMB remarkable flexibility and superior modeling capabilities compared to traditional ML methods, enabling it to simulate complex functions with fewer parameters, thereby enhancing model interpretability.

Applying KANMB to the metabolomic analysis of *S. alterniflora*, we demonstrated its superior performance in identifying metabolic biomarkers associated with plant salt stress responses. When benchmarked against other ML methods, including SVM, DL, XGBoost, and RF, KANMB more accurately identifies metabolic biomarkers linked to salt stress. Specifically, KANMB successfully pinpointed 226 significant metabolites with a high prediction probability threshold of 0.99 and further sifted through 5737 DEGs that are intimately associated with these metabolites. These DEGs were profoundly enriched in pathways closely related to salt stress responses in GO and KEGG analyses, such as glutathione metabolism, MAPK signaling pathway, and flavonoid biosynthesis. These discoveries unveiled crucial mechanisms underlying plant adaptation and response to salt stress. In contrast, while other ML methods also detected a certain number of metabolites and DEGs, their enrichment in salt stress‐related pathways was comparatively lower, indicating that KANMB excels in the precise localization and classification of metabolites.

### 3.2 The Complex Physiological and Biochemical Mechanisms of *S. alterniflora* in Salt Adaptation

Salt tolerance in *S. alterniflora* is governed by intricate regulatory mechanisms at both the transcriptional and metabolic levels. Genomic adaptations, including a higher prevalence of gene duplications and salt stress‐responsive genes, contribute to its enhanced salt tolerance.^[^ ^3^ ^]^ In this study, we observed that *S. alterniflora* exhibits a heightened number of DAMs and DEGs under high salt concentrations, manifesting as an increased count of DEGs, greater transcript diversity, and the activation of a wider array of pathways. Furthermore, salt stress promotes alterations in numerous metabolic pathways related to salt tolerance. Specifically, pathways such as ABC transporters, autophagy, and flavonoid biosynthesis, which have been previously reported to play crucial roles in stress responses,^[^ ^25^ ^]^ were significantly enriched in *S. alterniflora* under salt stress. Additionally, we identified salt stress‐associated GO categories like “oxygen‐containing compounds”, “active transmembrane transporter activity”, “transmembrane transporter activity”, and “transporter activity” that were enriched in *S. alterniflora* but not in non‐halophytic plants like rice and *Arabidopsis*.^[^ ^26^ ^]^ These genetic alterations significantly contribute to the robust salt tolerance displayed by *S. alterniflora*.

Metabolomic shifts induced by salt stress in *S. alterniflora* were also evident, particularly in the differential expression of flavonoids such as 3′,4′,5,7‐Tetrahydroxyflavone, Diosmetin, Quercetin 3,3′,4,5,7‐Pentahydroxyflavone, and Quercetin 3‐O‐sophoroside. GO and KEGG analyses of genes associated with these differential metabolites and biomarkers also revealed their extensive involvement in salt stress‐related processes or pathways. Notably, these findings contrast with observations in other halophytic plants like *Sesuvium portulacastrum* and non‐halophytic plants like rice and *Arabidopsis*.^[^ ^26^, ^27^ ^]^

Beyond molecular and metabolic adaptations, halophytes possess unique physiological structures that enable them to adapt to saline environments, distinct from those of non‐halophytic plants. One such feature is the salt gland structure in *S. alterniflora*, which continuously regulates ion transport during salt stress adaptation. Our study focused on leaf tissues due to their active role in salt secretion, though root tissues are also critical for ion uptake, osmotic regulation, and early stress signaling.^[^ ^21a^ ^]^ Future studies integrating root metabolomic and transcriptomic profiles will provide a more comprehensive understanding of whole‐plant salinity adaptation. Similar to *S. alterniflora*, the tissues of *Chenopodium quinoa* and *Mesembryanthemum crystallinum* exhibit abundant epidermal bladder cells.^[^ ^28^ ^]^ These structures can store significant amounts of salt, facilitating the coordination of ion transport in response to saline stress. Additionally, the tissue surfaces of *Limonium bicolor* are equipped with salt glands that expel excess salt from the plant's body, thereby maintaining ion homeostasis and preventing toxicity.^[^ ^29^ ^]^ The absence of such specialized structures in non‐halophytic plants likely contributes to their reduced salt tolerance.

Our findings demonstrate that *S. alterniflora* maintains robust physiological activity under 100 mm NaCl. Even at higher NaCl concentrations, it continues to thrive, showcasing its ability to self‐regulate through salt gland‐mediated ion homeostasis. This study underscores the interplay between genetic, metabolic, and physiological adaptations that collectively enable *S. alterniflora* to thrive in saline environments.

### 3.3 *SaMYB35* Enhances Plant Salt Tolerance by Regulating the Flavonoid Biosynthesis Pathway

MYB proteins frequently modulate the expression of downstream genes, thereby influencing the salt tolerance of plants.^[^ ^30^ ^]^ For instance, SiMYB16 from foxtail millet imparts salt tolerance in transgenic rice by modulating the phenylpropanoid pathway, with its overexpression significantly enhancing the accumulation of flavonoids, lignin, and the activity of fatty acid synthase in seedlings under salt stress conditions.^[^ ^31^ ^]^ Similarly, salt‐induced phosphorylation of GmMYB173 in soybeans leads to increased affinity for the *GmCHS5* promoter, thereby promoting *GmCHS5* transcription and enhancing salt tolerance through accumulated dihydroxy B‐ring flavonoids.^[^ ^32^ ^]^ Furthermore, VvMYBF1 actively regulates flavonol synthesis in grapes by controlling the expression of *VvFLS1*.^[^ ^33^ ^]^ In this study, we observed differential expression patterns of *MYB* and *bHLH* TFs in *S. alterniflora* under salt stress conditions, which led to identification of three MYB TFs and one bHLH TF. Notably, we discovered that the MYB TF, SaMYB35 could bind to the promoters of two *FLS* genes, *SA_13G035900.m1* and *SA_14G034400.m1*, and activate their expression. Subsequently, heterologous expression of *SaMYB35* in rice resulted in enhanced salt tolerance, supporting the role of elevated flavonoid content in conferring salt tolerance, and suggesting a potential strategy to improve adaptation to high salinity in other crops. These results demonstrate that MYB proteins confer resistance to plants by regulating the expression of various genes involved in biosynthetic pathways or altering flavonoid levels. To a certain extent, this underscores the role of *MYB* genes as a significant component within a vast salt tolerance regulatory system. Consequently, they emerge as potential targets for enhancing crop stress tolerance and quality through genetic engineering approaches.

## 4 Conclusion

In summary, our study introduced KANMB, a novel machine learning approach tailored for identifying salt‐responsive biomarkers in multi‐omics data. By applying KANMB to transcriptomic and metabolomic datasets from the halophyte *S. alterniflora* under varied salt conditions, we discovered key metabolic biomarkers, including those involved in flavonoid biosynthesis, crucial for salt tolerance. Overexpression of a key regulator *SaMYB35* in rice under high salinity enhanced flavonoid production and salt tolerance. Our study provides a genetic toolkit for breeding salt‐tolerant crops, showcasing machine learning's power in biomarker discovery and stress resilience, crucial for maintaining agricultural productivity in saline environments.

## 5 Experimental Section

### ML Approaches Identified Biomarkers

To accurately identify feature biomarkers, a ML model was developed, KANMB, which incorporated a framework comprising four base models (XGBoost, GBDT, RF, and SVM) along with a KAN network (Figure 1a). The four base models were initially built through hyperparameter optimization, with the following settings: the parameters for the XGBoost model were n_estimators = 701, max_depth = 9, and learning_rate = 0.02; the parameters for the RF model were n_estimators = 585 and max_depth = 15; the parameters for the SVM model were C = 0.233, kernel = “poly”, and degree = 3; and the parameters for the GBDT model were learning_rate = 0.178, n_estimators = 223, and max_depth = 5. To further enhance identification accuracy, a KAN network model was also trained, using the outputs of XGBoost, GBDT, RF, and SVM as a new feature matrix input for the KAN model. The KAN layer was composed of an input layer, a hidden layer, and an output layer, where each layer incorporates a set of learnable 1D learnable activation function. KANs’ nodes perform summation on the incoming signals of each spline function without applying additional nonlinear transformations, allowing the learned functions to adaptively represent the input features. In the study, the functional expression of the KAN model can be defined as:

(1) $$KAN\left(x\right)=\left(\phi_{l-1^{\circ }}\phi_{l-2^{\circ }}\cdot \cdot \cdot_{\circ }\phi_{1\circ }\circ \phi_{0}\right)\left(x\right)$$

where *x* is a new feature matrix comprised of the four base model output probability values, ϕ_*l*_denotes a set of learnable activation function of the *l^th^* KAN layer. In the study, four basic machine learning models were trained and evaluated as well as a KAN model through tenfold cross‐validation.

For comparison analysis, a DL model was also construed for biomarkers identification. The DL model designed for binary classification comprises six distinct layers (Figure 1b): (1) Input Layer, accepting a (36,) vector as raw data entry; (2) Normalization Layer, standardizing inputs for consistency across instances, though normalization specifics were not detailed; (3) First Fully Connected Layer with 32 neurons and *ReLU* activation, enhancing nonlinear relationship capture; (4) Second Fully Connected Layer, containing 16 neurons with *ReLU*, further processing features; (5) Third Fully Connected Layer, expanding to 32 neurons with *ReLU*, enabling more complex data representations; (6) Output Layer, a single neuron with Sigmoid activation, calculating the positive class probability. For efficient training, this approach was tailored with focal_loss to address class imbalance and Adadelta optimizer for dynamic learning rate adjustment, aiming to prevent overfitting. For optimization, Adadelta was chosen, dynamically adjusting the learning rate during training based on past parameter updates, negating the need for manual tuning. To guard against overfitting, early stopping and fivefold cross‐validation was incorporated. Early stopping halted training if validation loss stagnated for 50 epochs, while cross‐validation assessed the model's generalization ability by iteratively training and evaluating on different subsets of the data. The parameter configurations for other models were provided in (Table S9, Supporting Information).

### Plant Growth, Salt Treatment, and Ion Content Analysis

Seeds of *S. alterniflora* were collected from Dongying, Shandong Province, China (N37°26′; E118°34′). Germinated seedlings were transplanted into a nutrient‐rich substrate and allowed to grow for one week. Subsequently, the seedlings were irrigated with Hoagland's solution containing various concentrations of NaCl: 0, 100, 200, 300, 500, and 700 mM. The growth of the seedlings was continuously monitored, and on the 60th day, plant height measurements were taken. Samples were then collected for subsequent experiments. Specifically, the second leaf from the top was excised for transcriptome and metabolome analysis, as well as for the determination of ion content in the leaves. The underground portion of the plant was washed clean with water and used for ion content measurements. All samples were immediately frozen in liquid nitrogen and stored at ‐80 °C for further used.

For ion content determine, inductively coupled plasma mass spectrometer (ICP‐MS; SUPEC 7000 series, Hangzhou, China) was employed to measure the concentrations of ions (Na^+^, K^+^, and Ca^2+^) in 0.1 g of tissue samples from roots or leaves after salt stress treatment. Eight biological replicates were carried out for each treatment to facilitate the analysis. For the measurement of physiological parameters, chlorophyll content was precisely quantified using a SPAD‐502 chlorophyll meter (Konica Minolta). The enzymatic activities of SOD, POD, and CAT, as well as the quantities of MDA and proline, were accurately determined using specialized assay kits from Solarbio Life Science, following the manufacturer's precise instructions. The electric conductivity was measured using a conductivity meter, while the Pn was determined using a LI‐6400/XT photosynthesis system. Each sample underwent eight biological replicates to ensure reliability and accuracy.

### Metabolome and Transcriptome Profiling

For metabolome, the leaf samples of *S. alterniflora* were freeze‐dried utilizing a vacuum freeze‐dryer. Subsequently, a mixer mill (MM 400, Retsch) was employed to pulverize the samples, which were then dissolved in 1.2 mL of 70% CH_3_OH solution, containing 100 mg of lyophilized powder. This solution was vortexed for 30 seconds, repeated every 30 minutes for a total of six cycles. Following this, the samples were refrigerated overnight at 4 °C. The UPLC‐MS/MS analysis and bioinformatics analysis of secondary metabolites were conducted by Novogene Biotechnology Co., Ltd. (Beijing, China). Metabolites were considered differentially accumulated if they satisfied the criteria of having an |Log_2_FC| ≥ 1 and a Variable Importance in the Projection (VIP) ≥ 1.

For RNA‐seq, total RNA was extracted from leaf samples using the RNAprep Pure Plant kit (TIANGEN, Beijing, China). RNA purity and integrity were assessed using a NanoPhotometer spectrophotometer (IMPLEN, CA, USA) and a Bioanalyzer 2100 system (Agilent Technologies, CA). Approximately 3 µg of high‐quality RNA sample was used for sequencing library preparation. The resulting cDNA libraries from wheat leaves were sequenced on the Illumina sequencing platform based on RIN > 7.0. After quality control measures, including adapter sequence trimming and removal of low‐quality reads, high‐quality RNA‐seq reads from each library were aligned to the assembled *S. alterniflora* reference genome using TopHat v2.0.14.^[^ ^34^ ^]^ Gene expression values were calculated in terms of FPKM. Reads that uniquely mapped to reference sequences without mismatches were utilized in the subsequent analysis of differentially expressed genes (DEGs) using DESeq2 with thresholds set at |Log_2_FC| ≥ 1 and a false discovery rate (FDR)‐adjusted *P* < 0.01.^[^ ^35^ ^]^

To establish biologically meaningful correlations, a Pearson correlation coefficient (PCC) threshold of > 0.8 was applied to assess relationships between DEGs and metabolite accumulation.

### PCA Analysis and Clustering of Metabolomes and Transcriptomes

To compare the protein‐coding gene profiles and metabolite profiles of *S. alterniflora* under salt stress conditions, PCA and hierarchical clustering in R software was performed using the procomp function and ComplexHeatmap,^[^ ^36^ ^]^ respectively. The PCA analysis and hierarchical clustering utilized the *Z*‐score transformed and normalized accumulation values of genes and metabolites.

### Gene Ontology Annotation and KEGG Analysis

For gene annotation, DEGs were annotated utilizing the NetGO 3.0 protein language model,^[^ ^37^ ^]^ adhering to the methodology detailed in the source article. The annotations were subsequently categorized as being enriched in specific gene sets, based on a significance level of *P* < 0.05 (determined by Fisher's exact test) and an enrichment threshold of at least 1.5‐fold above the background level. To annotate and enrich all metabolites, the KEGG compound database and the KEGG pathway database were utilized,^[^ ^38^ ^]^ as well as the Human Metabolome Database^[^ ^39^ ^]^ and LIPID MAPS.^[^ ^40^ ^]^

### WGCNA

The gene co‐expression networks were constructed utilizing the WGCNA package in R version 4.0.0.^[^ ^41^ ^]^ By employing the blockwiseModules function with the specified parameters: power set to 8, TOMType set to unsigned, minimum module size of 30, mergeCutHeight of 0.25, and corType set to Pearson, the gene co‐expression modules were successfully obtained.

### TF Prediction and Promoter Identification

To identify TFs among the DEGs in *S. alterniflora*, each DEG was utilized as a query in a BLAST search against the PlantTFDB,^[^ ^42^ ^]^ with a threshold of *E*‐value < 10^−5^. Following this, the genomic DNA sequence located 2000 base pairs upstream of the start codon and employed PlantCARE^[^ ^43^ ^]^ was surveyed to analyze the presence of binding sites within the promoter region.

### Genetic Transformation of Rice and Salt‐Treated Rice

The coding region of *SaMYB35* was amplified using the pUBI‐*SaMYB35*‐PF and pUBI‐ *SaMYB35*‐PR primers and inserted into the plant expression vector under control of the UBI promoter to generate pUBI‐*SaMYB35* (Table S10, Supporting Information). The recombinant plasmid was transformed into *Oryza sativa* ZH11 by *Agrobacterium*‐mediated genetic transformation. The third‐generation homozygous lines were selected for subsequent experiments. For the rice salt treatment experiment, three‐week‐old seedlings were exposed to 100 mm NaCl for a duration of 7 days. Following the salt treatment, the survival rate was calculated, and leaves were harvested for subsequent gene expression analysis. Additionally, the flavonoid content was quantified in leaves (0.1 g) collected from both overexpression lines and ZH11 plants, which were three weeks old and grown under normal conditions. These plants were then subjected to a 7‐day stress treatment with 100 mm NaCl. The flavonoid measurements adhered to the protocols specified in the Flavonoid Assay Kit (Comin, LHT‐1‐G). To ensure accuracy, all experiments were conducted in triplicate.

### RNA Extract and Quantitative RT‐PCR (qRT‐PCR)

Total RNA was extracted from the leaves of *S. alterniflora* using Plant RNA Purification Reagent (TIANGEN, Beijing), adhering strictly to the manufacturer's instructions. Reverse transcription was then carried out utilizing the Evo M‐MLV kit from Accurate Biology (Changsha, China) to synthesize the first‐strand cDNA. Following this, the cDNA was diluted tenfold and employed as the template for quantitative real‐time PCR (qRT‐PCR) analysis. Specific primers utilizing Oligo 7.0 and conducted the qRT‐PCR were designed using the SYBR Green kit from Accurate Biology (Changsha, China), strictly adhering to the manufacturer's guidelines. Each treatment consisted of three biological replicates, and the relative expression levels were calculated using the 2^−ΔΔCt^ method.^[^ ^44^ ^]^ The data collected were analyzed and graphically represented using Excel 2010, the least significant difference (LSD) test was employed, with a significance threshold set at *P* < 0.05.

### Transient Dual‐Luciferase Reporter Assays in Protoplasts and Subcellular Localization

The full‐length coding sequences of TF genes were cloned into the pGreenII 62‐SK vector to produce effector gene expression constructs, adhering to the previously described methodology.^[^ ^45^ ^]^ Simultaneously, the promoter sequences of target genes were inserted into the pGreenII 0800‐LUC double‐reporter vector. Subsequently, both the reporter and effector plasmids were introduced into tobacco leaf protoplasts via polyethylene glycol 6000‐mediated transformation. The empty pGreenII 62‐SK vector served as the negative control. The results were presented as the ratio of LUC to REN activity, based on three biological replicates. Transient expression assays isolation of protoplasts and transient expression assays were conducted following previously described protocols of the previous study (Chen et al., 2024). For subcellular localization, SaMYB35 coding sequences were amplified from NaCl‐treated *S. alterniflora* seedlings using primers pAN580‐SaMYB35‐PF and pAN580‐SaMYB35‐PR. The fragment was cloned into the pAN580 vector, which carries the CaMV *35S* promoter and an enhanced green fluorescent protein (eGFP) ORF. Protoplasts isolated from 7‐day‐old rice leaf sheaths were transformed with recombinant plasmids and incubated at 28 °C for 16 h. Green fluorescence was observed under a Zeiss LSM880 confocal microscope.

### Y1H Assay and Yeast Expression Analysis

For the Y1H assay, the 2‐Kb promoter DNA of the two *FLS* genes was cloned into the pAbAi vector, resulting in the creation of the bait construct. The full‐length coding sequences of *SaMYB35* was fused to the GAL4 activation domain in the pGADT7 vector, generating the prey construct *SaMYB35*‐pGADT7. The prey vector and the empty vector, serving as the negative control, were separately transformed into yeast cells that already contained the bait constructs. The transformed yeast cells were then diluted in a tenfold serial dilution and spotted onto SD medium plates lacking Leu and Ura, supplemented with 200 ng/ml Aureobasidin A (AbA). The interaction between the prey protein and bait sequence was assessed by monitoring the growth of yeast cells that harbored both the bait and prey constructs. For yeast expression analysis, the CDS of *SaMYB35* was cloned into pYES2 and transformed into INVSc1 yeast cells. Phenotypic analysis involved culturing transformed yeast in media with either 0 mm or 100 mm NaCl at 29 °C for three days, after which were imaged and analyzed.

### Phylogenetic Analysis

Protein sequences from diverse species were retrieved from the NCBI. These sequences were then aligned using T‐COFFEE.^[^ ^46^ ^]^ Following alignment, the data was employed to construct a phylogenetic tree in MEGA 7,^[^ ^47^ ^]^ adopting the neighbor‐joining (NJ) method with 1000 bootstrap replicates for increased confidence in the tree topology.

## Conflict of Interest

The authors declare no conflicts of interest.

## Author Contributions

H.H.L. conceived the original idea, designed the experiments, and provided overall supervision of the work. S.K.C., H.Z., S.Q.G., K.H.H., and T.X.Y. performed the experiments and data analysis. S.G. and J. W. provided valuable advice for the experimental design and optimization. S.K.C. and H.H.L. wrote the manuscript. All authors discussed and commented on the manuscript.

## Supporting information



Supporting Information

Supplemental Table 1

## Data Availability

The data that support the findings of this study are available in the supplementary material of this article.
