# Supplementary material for: Unveiling Salt Tolerance Mechanisms in Plants: Integrating the KANMB Machine Learning Model With Metabolomic and Transcriptomic Analysis
Source: Adv Sci (Weinh). 2025 Apr 26;12(23):2417560. doi: 10.1002/advs.202417560 (PMC12199390; doi:10.1002/advs.202417560)
Supplement: Supplementary file 1 — Supporting Information [file ADVS-12-2417560-s002.docx]

**Supplementary Information**

**Unveiling Salt Tolerance Mechanisms in Plants: Integrating the KANMB Machine Learning Model with Metabolomic and Transcriptomic Analysis**

*Shoukun Chen^1,2^, Hao Zhang^1,2^, Shuqiang Gao^1,2^, Kunhui He^1,2^, Tingxi Yu^1,2^, Shang Gao^1,2^, Jiankang Wang^1,2^, Huihui Li^1,2,*^*

**Supplementary Figures 1-8**

**
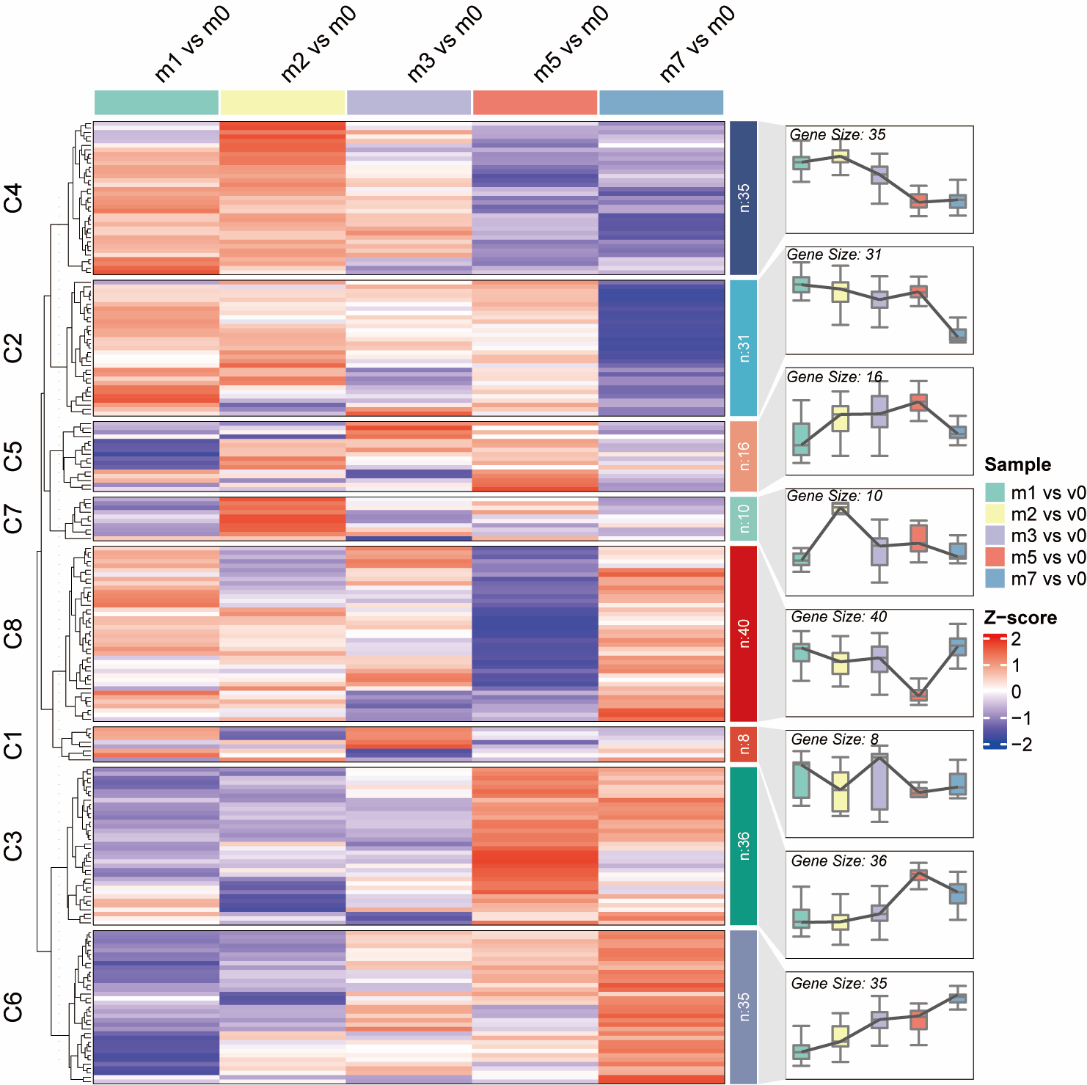
**

**Figure S1. Trend expression analysis of differentially expressed genes (DEGs), clustered into eight distinct groups.**


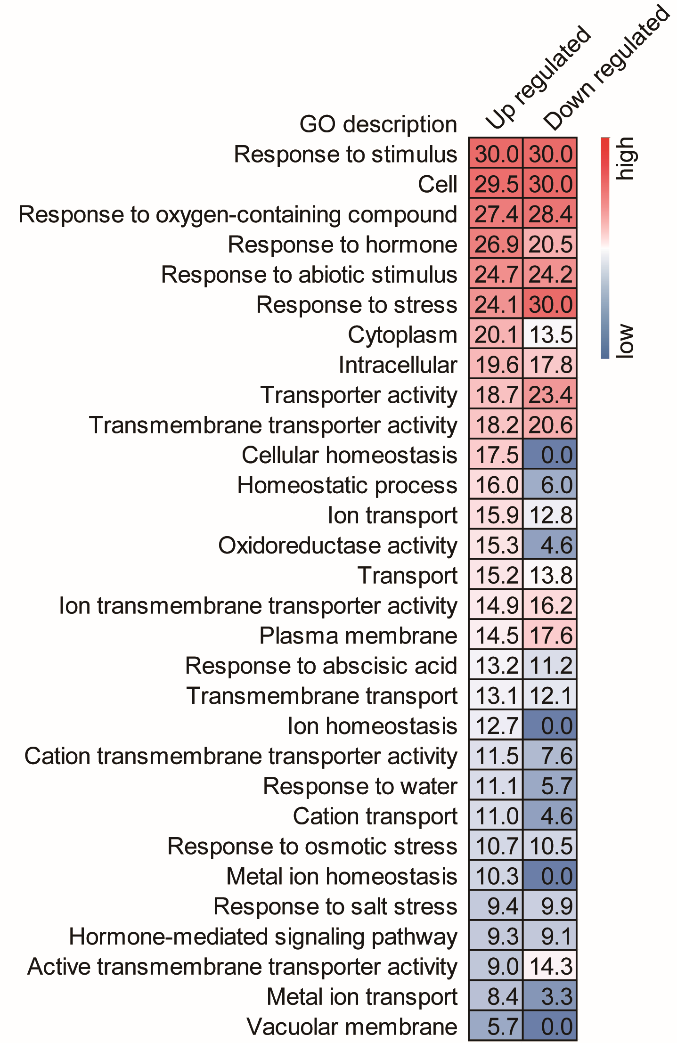


**Figure S2.** **GO analysis of all differentially upregulated and downregulated genes under salt stress treatment.**


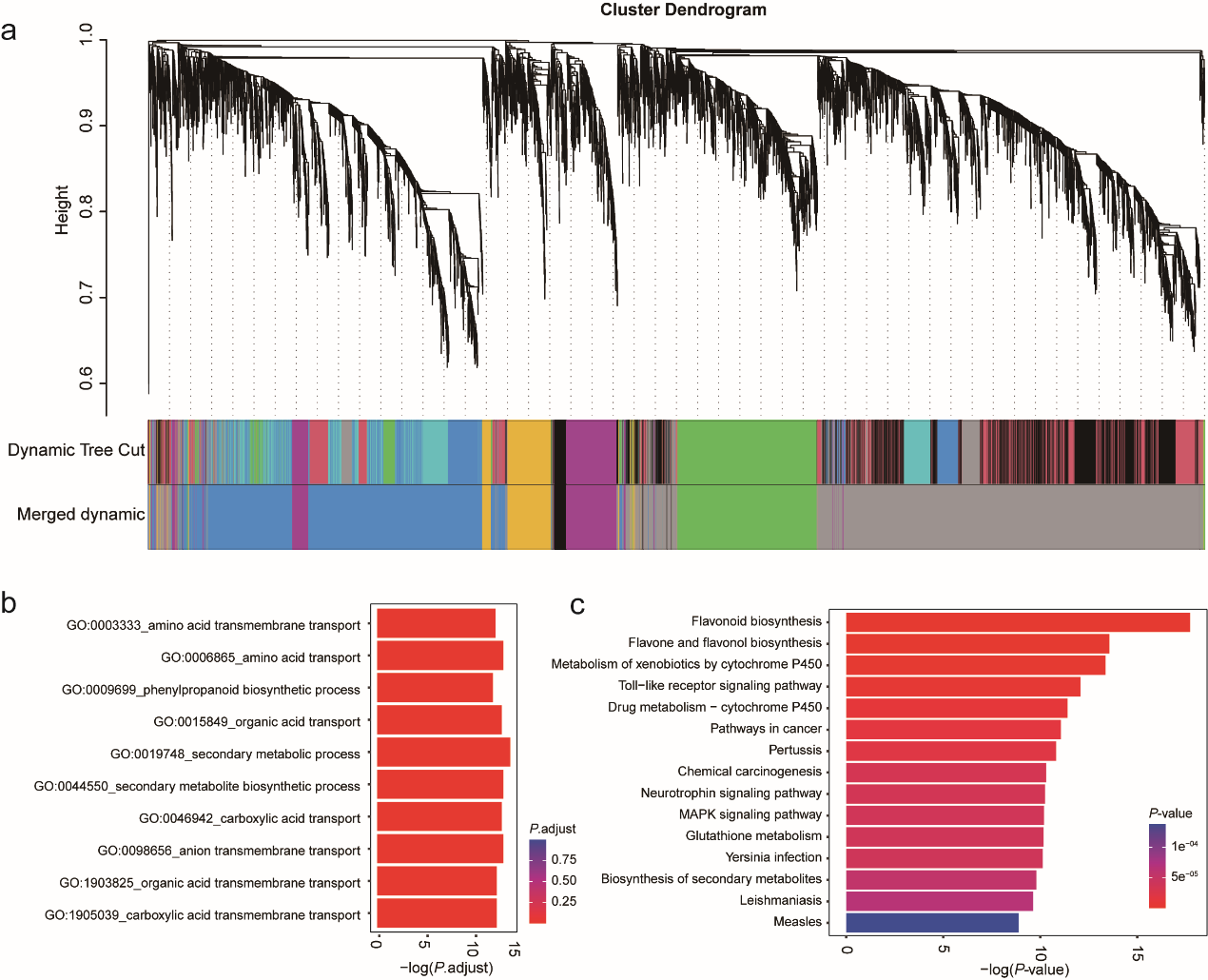


**Figure S3. Weighted gene co-expression network analysis of the transcriptome of *S. alterniflora* in response to salt stress.** (a) The nine gene co-expression modules revealed by hierarchical cluster tree and heatmap. (b) GO annotation of genes in the gray module; (c) KEGG analysis of genes in the gray module.

**Figure S4**. **Comparative performance of five models regarding prediction accuracy, recall, and AUC.**


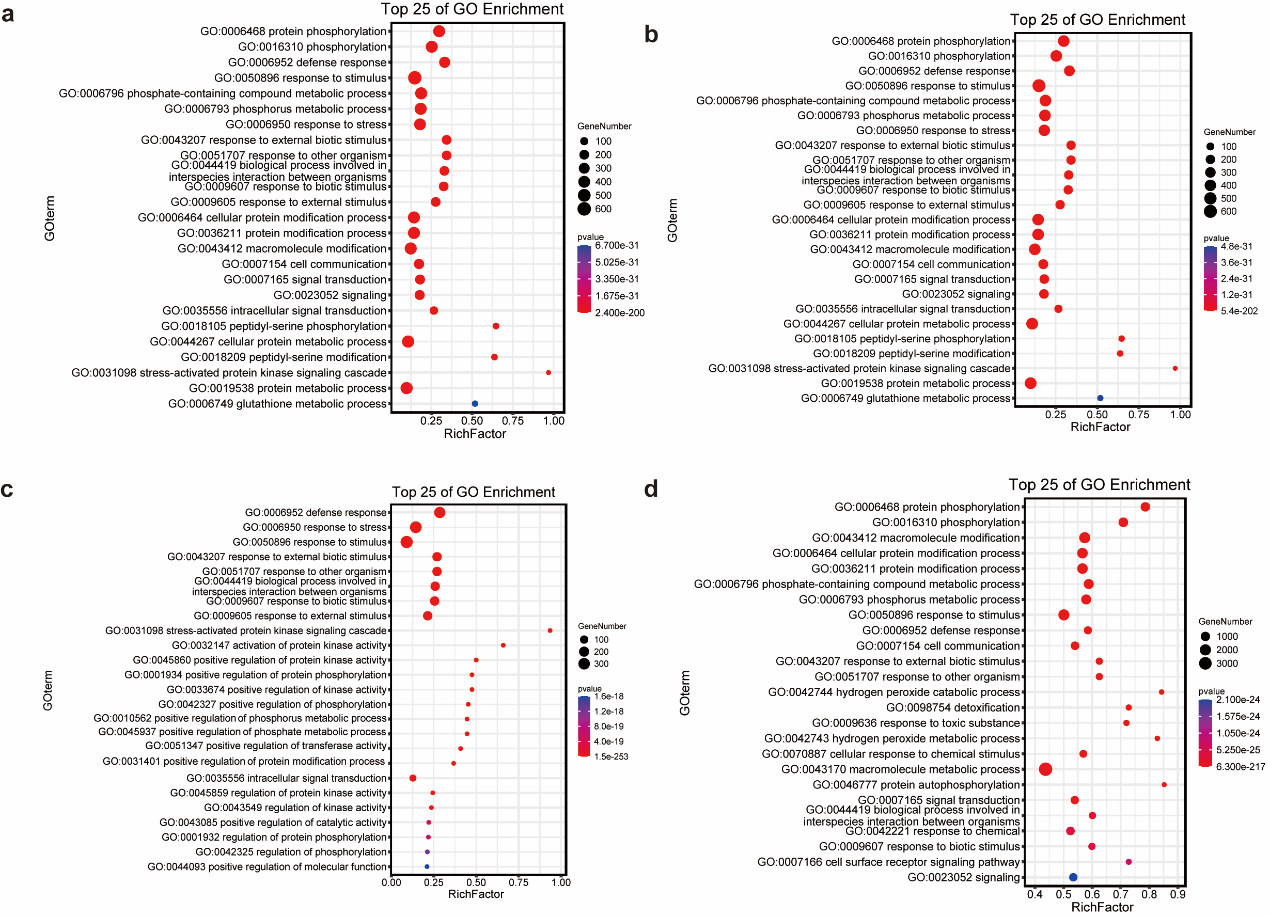


**Figure S5. GO analysis of DEGs corresponding to the biomarkers identified by (a) SVM, (b) DL, (c) XGBoost, and (d) GBDT.** The Figure displays only the top 25 GO enrichment categories for the DEGs associated with each machine learning method.


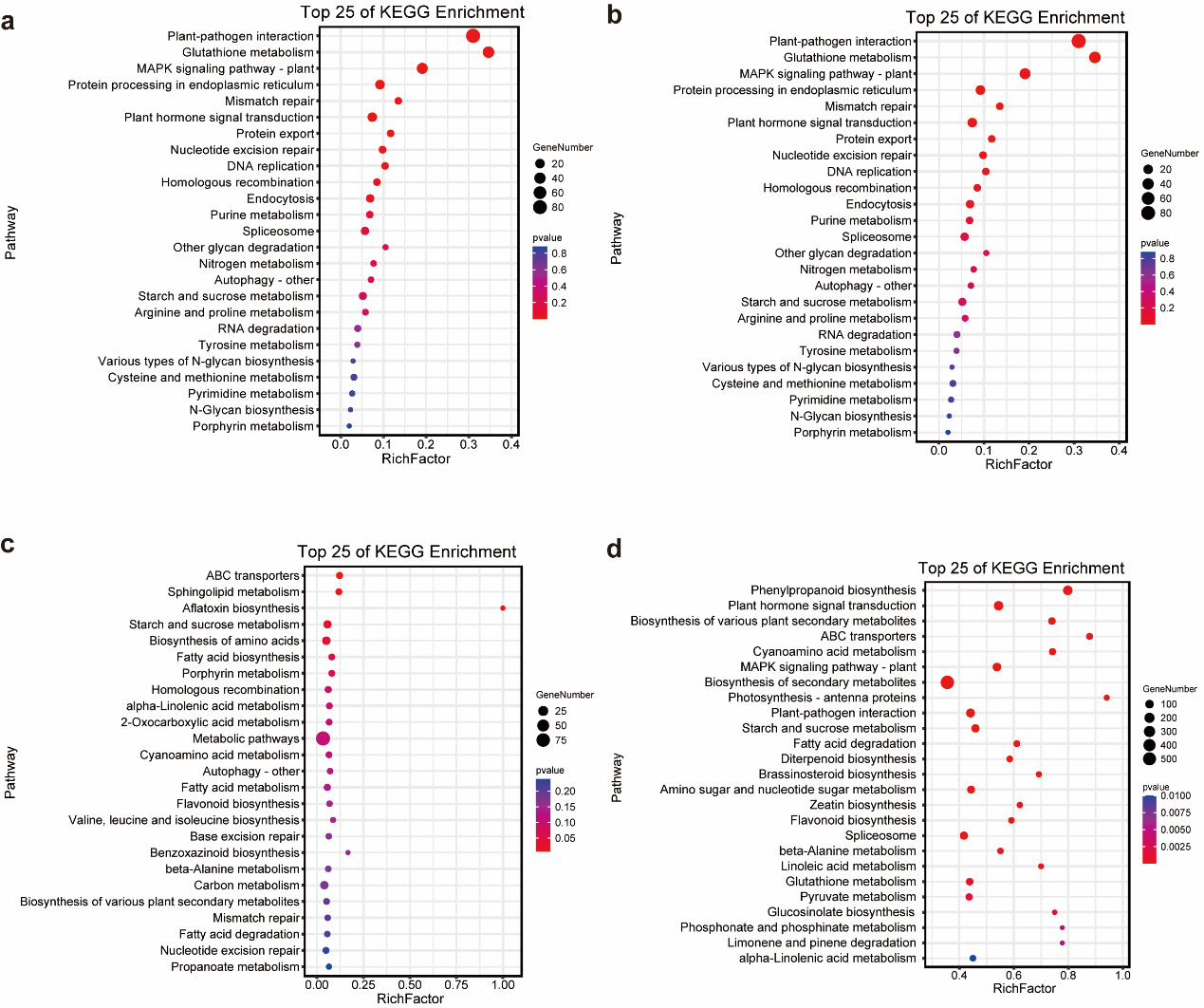


**Figure S6.** **KEGG analysis of DEGs associated with the identified biomarkers.** In the figure, a, b, c, and d represent the KEGG enrichment results of DEGs obtained through three machine learning methods: SVM, DL, XGBoost, and GBDT, respectively. Only the top 25 enriched KEGG categories for the DEGs corresponding to each machine learning method are displayed in the figure.


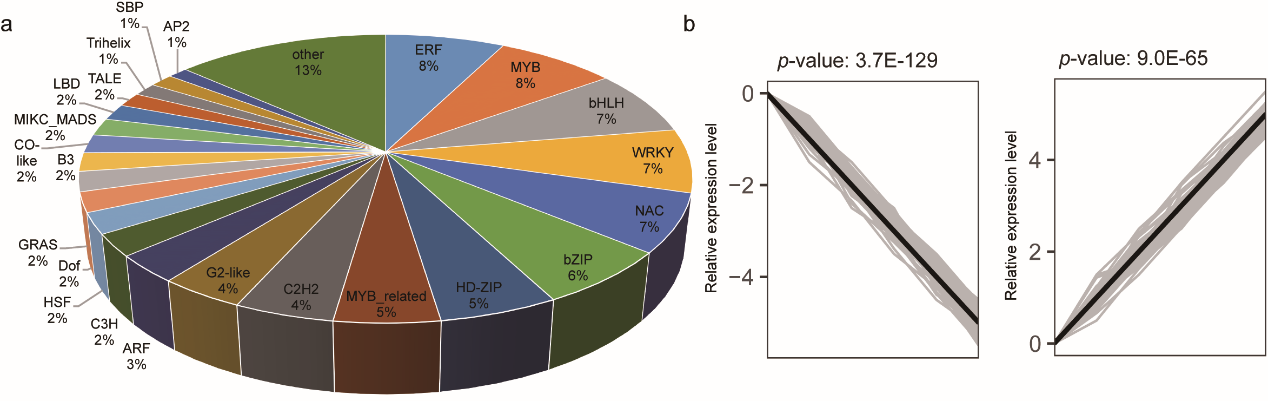


**Figure S7**. **Cluster analysis of co-expression patterns between flavonoid biosynthesis enzyme genes and all differentially expressed transcription factors after salt stress treatment.**


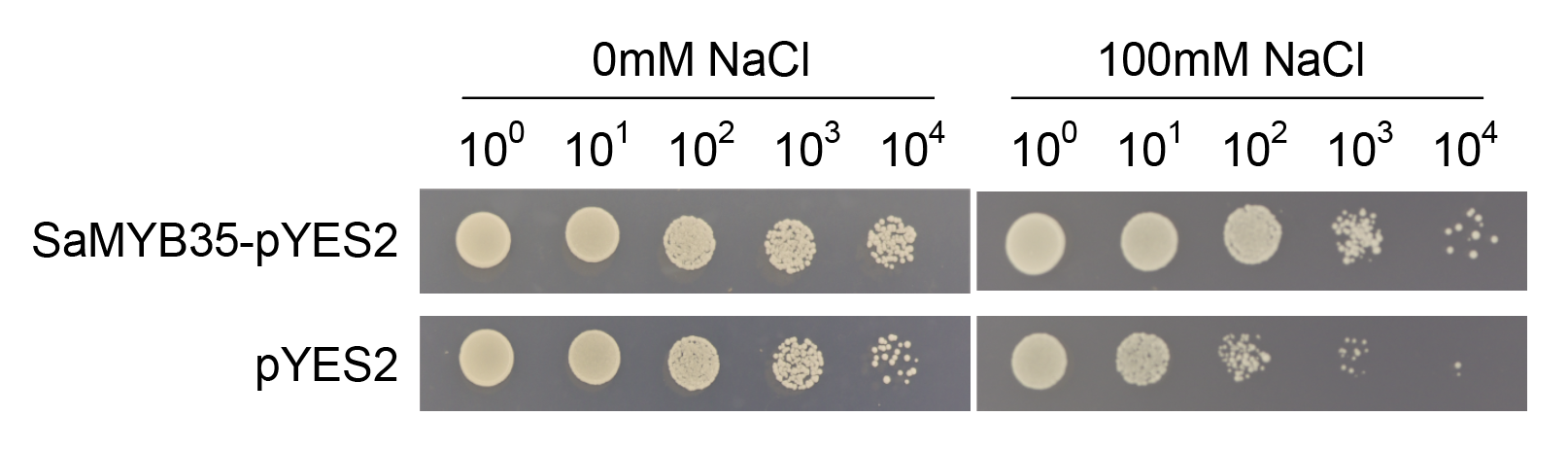


**Figure S8. Phenotypic effects of different NaCl concentrations on yeast cells expressing *SaMYB35*.** Yeast cells were transformed with either the pYES2 vector (control) or SaMYB35-pYES2 and subjected to 10-fold serial dilutions before being spotted onto SD-URA plates with 0 or 100 mM NaCl. Plates were incubated at 29 °C for three days before imaging.
